# A Low Concentration of Ethanol Impairs Learning but Not Motor and Sensory Behavior in Drosophila Larvae

**DOI:** 10.1371/journal.pone.0037394

**Published:** 2012-05-18

**Authors:** Brooks G. Robinson, Sukant Khurana, Jascha B. Pohl, Wen-ke Li, Alfredo Ghezzi, Amanda M. Cady, Kristina Najjar, Michael M. Hatch, Ruchita R. Shah, Amar Bhat, Omar Hariri, Kareem B. Haroun, Melvin C. Young, Kathryn Fife, Jeff Hooten, Tuan Tran, Daniel Goan, Foram Desai, Farhan Husain, Ryan M. Godinez, Jeffrey C. Sun, Jonathan Corpuz, Jacxelyn Moran, Allen C. Zhong, William Y. Chen, Nigel S. Atkinson

**Affiliations:** Section of Neurobiology, Institute for Neuroscience, The University of Texas at Austin, Austin, Texas, United States of America; Center for Genomic Regulation, Spain

## Abstract

*Drosophila melanogaster* has proven to be a useful model system for the genetic analysis of ethanol-associated behaviors. However, past studies have focused on the response of the adult fly to large, and often sedating, doses of ethanol. The pharmacological effects of low and moderate quantities of ethanol have remained understudied. In this study, we tested the acute effects of low doses of ethanol (∼7 mM internal concentration) on Drosophila larvae. While ethanol did not affect locomotion or the response to an odorant, we observed that ethanol impaired associative olfactory learning when the heat shock unconditioned stimulus (US) intensity was low but not when the heat shock US intensity was high. We determined that the reduction in learning at low US intensity was not a result of ethanol anesthesia since ethanol-treated larvae responded to the heat shock in the same manner as untreated animals. Instead, low doses of ethanol likely impair the neuronal plasticity that underlies olfactory associative learning. This impairment in learning was reversible indicating that exposure to low doses of ethanol does not leave any long lasting behavioral or physiological effects.

## Introduction

Ethanol consumption is known to affect sensory and motor abilities and to compromise more complex cognitive functions, such as attention, learning, and memory. These effects are dose dependent and sensitive to heterogeneity in ethanol metabolism, body weight, gender, genetic background, and prior experience with ethanol. Cognitive tests have established that low amounts of ethanol disrupt attention-requiring tasks including learning and memory, while higher amounts of ethanol disrupt sensory and motor responses [1]–[3]. Many studies in animal models have focused on obviously intoxicating levels of ethanol that produce motor defects [4]–[7]. The effects of low doses of ethanol are understudied in animal models because the behavioral consequences are subtle. However, society incurs significant cost from accidents while operating machinery or driving at the levels of ethanol that affect judgment and attention in the absence of obvious effects on motor coordination [8], [9].

Ethanol has previously been shown to have many effects on learning and memory. In humans, ethanol disrupts performance on a variety of short-term memory tasks, from verbal list learning [10]–[12] to spatial memory [13]. Model systems have also been used to gain an understanding of the mechanisms behind ethanol-associated behaviors [4], [14]–[17]. In rodents, acute doses of ethanol have been shown to impair many learning tasks, including spatial memory [18]–[21], nonspatial working memory [22], [23], and spatial reference tasks [24]. In honeybees, consumption of a 5% or higher ethanol solution disrupts Pavlovian conditioning [25], [26]. Adult *Drosophila melanogaster* have been a particularly useful model organism for the genetic analysis of ethanol responses because they show many of the same responses to ethanol as do humans and, in addition, have the most experimentally malleable genome of any metazoan [16], [27]. As observed in mammals, flies become hyperactive when initially exposed to ethanol but suffer incoordination and sedation as their internal ethanol concentration rises [28]. Furthermore, like mammals, flies acquire functional tolerance to ethanol intoxication [29]–[31]. To date, the analyses of the effects of ethanol in Drosophila have been mostly restricted to the study of locomotor impairment. There is a need for a genetic model system to dissect how small amounts of ethanol affect emergent properties of the nervous system.

Adult flies have long been used to study behavioral plasticity, but over the last few years, larval Drosophila have become valuable as a genetic model for the study of learning and memory [32]–[36]. With a powerful genetic toolbox and a simple nervous system that generates a variety of behaviors, larvae are an excellent choice for genetic analysis of neural plasticity. Most ethanol-related studies in larvae have focused on ethanol preference and metabolism [37]–[42]. One recent study examined the effects of larval ethanol exposure on adult development and ethanol responses [43]. The natural habitat of larvae includes fermenting fruits that contain significant ethanol content [44], and it is likely that ethanol has significant impact on the physiology and behavior of larvae. Nevertheless, there has not been an in-depth study of the acute effects of ethanol on larval behavior.

In this study, we establish early third instar larvae of *Drosophila melanogaster* as an animal model to probe how small doses of ethanol affect learning, a higher order emergent property of the nervous system, while leaving the sensory and motor functions intact. We use heat-shock conditioning, a robust larval associative learning paradigm [32], to explore the effect of low pharmacologically relevant doses of ethanol on learning in *Drosophila melanogaster* larvae.

## Materials and Methods

Morphologically, larvae have three instars or stages, but the third and most advanced instar is functionally subdivided into two substages; an early third instar and a wandering late third instar [45]. The olfactory paradigm and conditioning protocol used in this study [32] were established for early third instar larvae.

### Fly Husbandry and Harvesting of Larvae

Wild type flies of the Canton S strain (Bloomington stock # 1) were raised on standard cornmeal/yeast/molasses media on a 12/12 light/dark schedule at 24°C. To produce age-matched early 3^rd^ instar larvae, adult flies were allowed to lay eggs on the media for 24 hours and then removed. Five days (+/− 8 hours) later, early 3^rd^ instar larvae were collected by dissolving the larvae-containing media in water and rinsing away softer media through a sieve that does not retain the smaller second instar larvae. Larvae were then placed in a 1500 molecular weight polyethylene glycol (PEG, Acros Organics AC19226-0051) solution that acts as a density separator wherein the larvae float and dense food particles sink. After the density separation, larvae were rinsed in water to remove traces of PEG. Larvae were then rested in 9 cm Petri dishes containing 0.5 ml of Ringer's solution until the onset of the experiment. The Ringer's solution contained 128 mM NaCl, 4.7 mM KCl, 1.8 mM CaCl_2_, 0.9 mM Na_2_HPO_4_, and 0.37 mM KH_2_PO_4_.

### Ethanol Treatment

**Figure 1:**
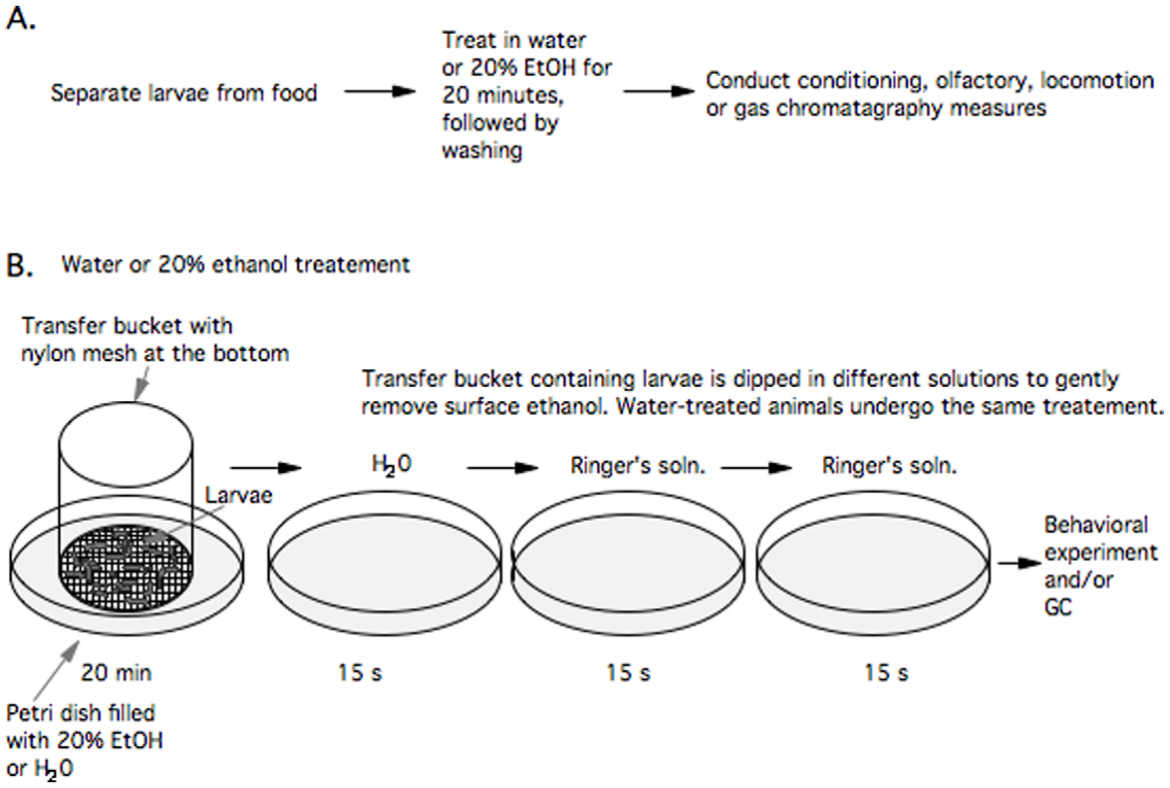
Experimental design. **A**. Schematic of the flow of the experiment. **B**. Schematic of the ethanol/water treatment protocol.

Larvae were treated with ethanol (Figure 1A) to determine if the ethanol affected their odor response or their associative learning capabilities. One hundred larvae were placed in a transfer chamber that was resting in a 5 cm diameter Petri dish containing 3 ml of 20% ethanol v/v diluted in water. The transfer chamber was a plastic beaker with the bottom replaced with a nylon mesh. Up to six groups of 100 larvae were treated simultaneously (see Larval Training and Testing below). Larvae were treated with ethanol for 20 minutes, rinsed with water and then Ringer's solution. Additionally, a group of larvae was treated with water only to serve as a control for the ethanol-treated group. Both ethanol- and water-treated animals underwent the same duration (20 minutes) of ethanol or water treatment and the same duration (45 seconds) of rinsing (See Figure 1B). After the ethanol or water treatment and subsequent rinsing, larvae were taken through one of the behavioral tasks: olfactory conditioning, olfaction, locomotion or heat avoidance.

### Associative Conditioning Apparatus and Setup

The experimental set up was similar to a previous study [32]. In associative conditioning, a conditioned stimulus (CS) is paired with an unconditioned stimulus (US) to produce an altered or conditioned behavioral response to the CS. In our heat shock learning paradigm, 10^−4^ dilution of the attractive odor ethyl acetate (EA, Fisher Scientific E145-1) was used as the CS and heat was used as the aversive US. The behavioral response following the pairing of these two stimuli is seen as a decrease in attraction to the EA. The presentation of the CS and US was done using agar-filled 9 cm glass Petri dishes. Specifically, 30 ml of 0.5% agar was poured into the Petri dishes. For heat presentation, the Petri dish was rested atop a heat block (Analog Dry Block Heaters VWR 12621) for 8 minutes prior to use. At equilibrium, the temperature of the top agar is 2°C lower than the temperature of the heat block. The temperature of agar surface was monitored in all experiments. Agar surface temperatures of 41°C and 35°C were used for training. For odor presentation, pure EA was mixed into the 0.5% melted agar just prior to solidification and vigorously shaken. Heating a Petri dish with odor mixed in allowed the simultaneous presentation of odor and heat. During the training, larvae were kept in a transfer chamber that was made by replacing the bottom of a plastic beaker (50 ml Tri-pour beaker no. 50-996-322, Fischer Scientific) with fine nylon mesh (obtained from a local fabric store). The transfer chamber allowed the experimenter to quickly move the larvae to different experimental conditions.

### Larval Training Procedure

Larvae were put in the transfer chamber and placed on a Petri dish filled with 0.5% agar at room temperature. To administer a heat shock, the transfer chamber was dipped in Ringer's solution, and then placed on a heated agar Petri dish. After a 30 second shock, the chamber was again dipped in Ringer's and placed back on the rest dish. An interval of 8 minutes was used between heat shocks. The training consisted of 3 shocks and the larvae were tested for their olfactory attraction within 5 minutes of the final shock. We confirmed that, regardless of the ethanol or water treatment, larvae exposed to the CS or the US alone had the same olfactory responses as sham-conditioned animals. Because of this, we used one control group for the remainder of the study, the heat only stimulus (US), in addition to the trained group that received the simultaneous odor-heat (CS-US) pairing.

### Larval Olfactory Testing

Following the training procedure, larvae were tested for their olfactory attraction to EA [32]. Olfactory testing was done on 15 cm Petri dishes containing ∼15 ml of 2% agar. 30 larvae were placed in the center of the plate in a zone of 1 cm radius. On one side of the plate, 20 µl of EA diluted in pure liquid paraffin to a concentration of 10^−4^ v/v was spotted onto a paper disc. The odor was spotted 3 cm from the edge of the plate. Diametrically opposite to the odor, liquid paraffin was spotted similarly. Larvae were allowed to roam the plate freely for 3 minutes, at which point the number of larvae in a 2 cm radius zone around the odor was noted as well as the total number of larvae on the plate. We also noted the number of larvae in a 2 cm radius zone opposite the odor zone. No attraction to the solvent alone was observed. Larvae that remained within the 1 cm drop zone were not counted because their lack of movement could be due to poor health. The number of these non-participants did not exceed 5% in any experiment. For each test plate, a response index (RI) was calculated as the fraction of total participating larvae on the plate that were found in the odor zone at the end of 3 minutes. For each experiment repetition, a minimum of 3 test plates were performed and averaged to give a single response index. For learning experiments, the response indices of the control group and trained group were compared to give a learning index. The learning index was defined as (RI_control_−RI_conditioned_)/RI_control_ and it represents the decrease in response to the odor caused by the training.

### Tracking Larval Olfactory Response

Larval tracking was done in a manner similar to a previous study [46]. Twenty-four hours prior to the start of the experiment black food dye was added to the larvae-containing food. The dyed food inside the larvae is easily visible due to the transparent larval body wall. Using a standard olfactory testing procedure (described above), a camera was placed above the Petri dish and captured a frame of the plate each second. The particle counting algorithm applied a binary threshold to each frame, so only pixels darker than the threshold were counted. These pixels were then grouped together into objects, and each object larger than three pixels was counted as a larva. We manually verified that the 3-pixel threshold was sufficient to capture over 99% fully separated larvae without capturing erroneous noise. The algorithm then calculated the coordinates of the centers of each larva. Using these coordinates, the algorithm then calculated the distance of each larval object to the odor center, which was predetermined. A Larval object that was less than 2 cm from the odor was counted as in the odor zone, and otherwise outside the odor zone. A correction factor had to be applied to each frame to account for the fact that individual larvae cannot be resolved in the very beginning of the test, when they are all aggregated in the center of the plate, and near the end of the test when the larvae are aggregated near the odor. The correction factor was as follows: for each frame of the movie, the number of larvae tracked was reported. The frame number that had the highest larval count was noted (and manually verified). For each frame prior to the highest larval count frame, the difference between the highest larval count and the count at that frame was assumed to be larvae in the center of the plate, i.e., outside the odor zone. For each frame after the highest larval count frame, the difference was assumed to be larvae inside the odor zone. After the correction factor was applied, the algorithm then calculated the number of larvae inside the odor zone and outside the odor zone. The response index was then calculated as described above. These steps were applied to each frame in the movie, so a response index vs. time plot could be generated.

### Gas Chromatography

Approximately 100 larvae were placed in 750 µl of pure toluene in a micro centrifuge tube immediately following the ethanol treatment and rinse. The weight of larvae was determined by weighing the centrifuge tube before and after adding the larvae. We determined the water content of larvae to be 81.4% of their weight by weighing larvae before and after desiccation in a 65°C oven. The larvae were crushed with a small pestle and the supernatant was removed after spinning the tube at 15 K rpm for 2 minutes. An auto sampler injected 3 µl of the extract into an SRI-310C Gas Chromatograph (SRI Instruments, Torrance, CA). The temperature protocol was: 50°C for 1 minute, ramp for 10 minutes to 150°C, and hold for 10 minutes. An ethanol peak is observed at approximately 2.2 minutes and toluene at approximately 10 minutes. All data were analyzed using PeakSimple (SRI Instruments, Torrance, CA). The area of the ethanol peak was determined using the integration tool with a threshold area size of 100. The ethanol content of the larvae was determined by a comparison to a known standard curve of ethanol. The concentration of ethanol in the larvae was determined by calculating the total number of mmoles of ethanol extracted from the larvae and dividing this by the total water content of the larval sample.

### Heat Avoidance assay

Larvae were tested for their sensitivity to heat or cold, in a manner adapted from Rosenzweig *et al.* [47], to ensure that effects on learning were caused by psychopharmacological properties of ethanol rather than anesthetic properties. To test heat sensitivity, a 9 cm glass Petri dish filled with 2% agar was situated on a heat block so that half of the plate was on the heat block and half of the plate was not. To test the cold sensitivity the agar plate was situated half on a heat block that had been cooled by ice to 18°C. In all, we tested the two temperatures used for training, 41°C and 35°C, as well as 31°C, 28°C, 26°C and 18°C to explore the sensitivity of the assay. Thirty larvae were placed in the center of the plate and at every 1-minute interval, for a total of 6 minutes, the number of larvae on each half of the plate was noted. An avoidance index was calculated by subtracting the number of larvae on the heated or cooled side from the number of larvae on the room temperature side and dividing the difference by the total number of larvae on the plate. In this index a negative score indicates attraction.

### Statistics

Animals that were trained together were considered to be the same sample (N = 1) due to a lack of independence despite the fact that multiple testing plates were used to evaluate learning. The learning index for each sample was the average of a minimum of three testing plates of 30 animals each. Error bars presented throughout the study are the standard error of the mean (SEM). The significance score was calculated using Students *t*-test for pair-wise two-tailed comparison. The number of experiments and p-values are stated in the results section and figure legends. In the figures we use “*” to indicate significance level <0.05 and >0.01, and “**” for p-values<0.01. For all single comparisons we present the exact p value and for multiple pairwise comparisons we present p values as lower or greater than 0.05.

## Results

We wished to determine whether low-level ethanol intoxication affects the capacity for learning in *Drosophila melanogaster* larvae. We define a low dose as a dose that does not produce obvious changes in locomotor activity nor blocks sensation. Larvae were separated from the media, treated with ethanol or water, and then taken through a behavioral test (Figure 1A). After the water or ethanol treatment we measured larval locomotion, olfaction, olfactory conditioning, and heat avoidance. Given that we wanted to correlate the behavior with the amount of ethanol in larvae, we also assessed larval ethanol content using gas chromatography. Additionally, we evaluated if the changes in animal behavior produced by ethanol are temporary by testing whether larvae recover a normal conditioning response after the ethanol has been metabolized.

### Larvae readily absorb low concentrations of ethanol and retain it for the duration of the learning assay

**Figure 2:**
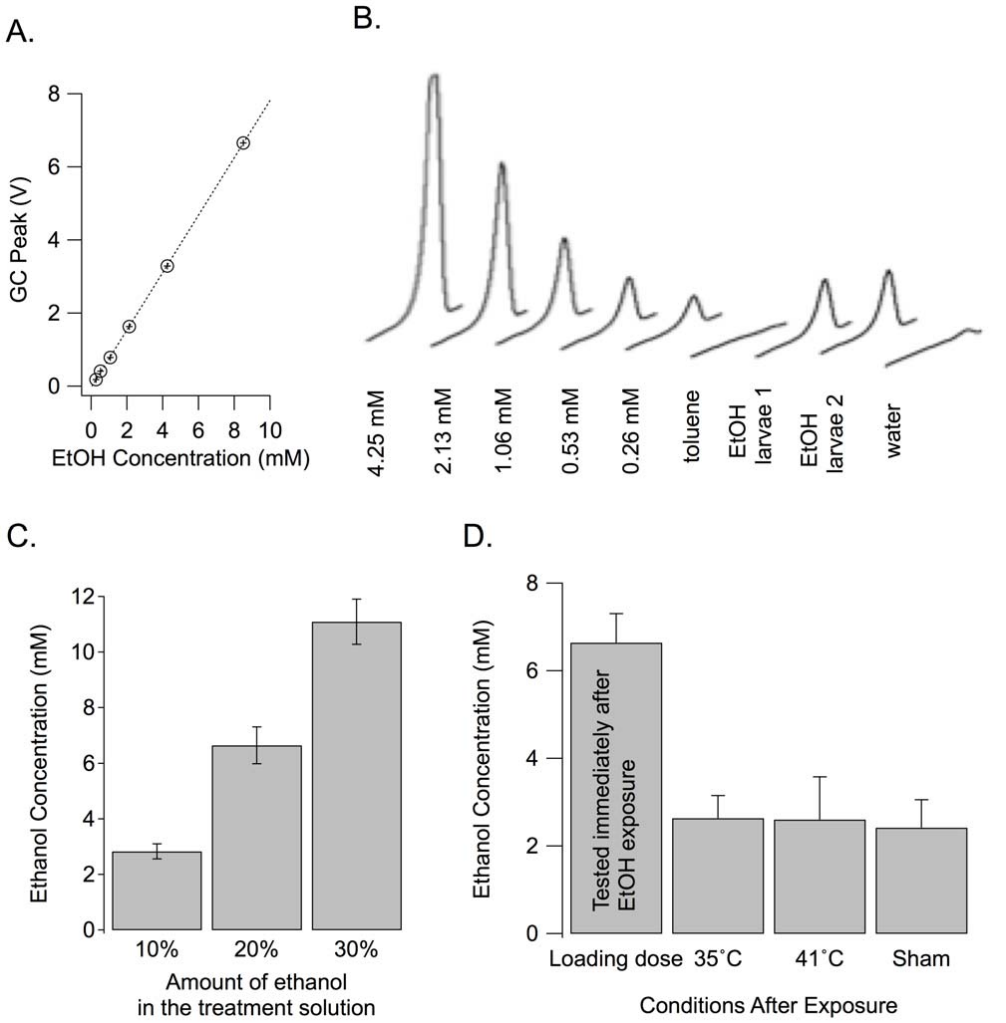
Perdurance of internal ethanol. **A**. Example standard curve for ethanol gas chromatography. All of the measurements noted in this document fall within the linear range of the gas chromatograph standard curve. **B**. Chromatographs of ethanol from larvae. Standard responses for known concentrations of ethanol (4.25 mM, 2.13 mM, 1.06 mM, 0.53 mM, and 0.26 mM) diluted in toluene as well as pure toluene are shown. Representative traces from larvae treated for 20 minutes with 20% ethanol (EtOH larvae 1 and 2) or water (control larvae) are also shown. **C**. The amount of ethanol absorbed by larvae depended on the amount of ethanol in the treatment solution. **D**. The brief heat shocks (41°C and 35°C) that were used in the conditioning experiments did not reduce internal ethanol below that measured in sham-treated larvae. Animals were treated for 20 minutes with 20% ethanol (Loading Dose) and then taken through the heat shock protocol at 35°C or 41°C as used in conditioning experiments. The loading dose is the same data shown in the panel C 20% bar graph and is repeated for comparison purposes. Sham-treated animals were taken through same protocol except that they did not receive the heat shocks but instead were moved to room temperature (24°C) plates.

We used gas chromatography to measure internal ethanol concentration in the larvae. Larvae were crushed into toluene, and compared to a known standard curve of ethanol in toluene. We used a large volume of toluene (750 µl) in which to crush approximately 100 larvae to ensure that the metabolic processes of the larvae were completely and abruptly stopped and to ensure that the ethanol in the larvae directly enters the solvent. Ethanol was clearly detectable in the larvae and within our standard curve (Figure 2A–B). Figure 2B shows an example trace from two larval groups that were exposed to 20% ethanol for 20 minutes, one water-exposed larval group, and a standard curve. We used the weight of the larvae to determine the internal ethanol concentration (larvae are ∼81% water by weight, see methods for details). We found that larvae absorb ethanol in a dose dependent manner (Figure 2C). Immediately after ethanol exposure, the internal ethanol concentration of larvae treated for 10 minutes with 10%, 20%, and 30% ethanol v/v diluted in water was 2.8±0.3 mM (n = 8), 6.6±0.7 mM (n = 13), and 11.1±0.8 mM (n = 8), respectively. No ethanol was detected in water-treated larvae (detection threshold ∼0.5 mM, data not shown). We also observed that significant ethanol is retained through the entire conditioning experiment (Figure 2D). Given the volatile nature of ethanol and that conditioning involves heat exposure, we measured ethanol decay after taking larvae through the exact conditioning protocol. We showed that, regardless of heat-shock temperature, 3 heat shocks of 30 s did not cause a significant change in the clearance of ethanol content compared to larvae not receiving heat shocks (n = 13 for “Loading Dose”; n = 5 for all others; p = 0.98, 0.79 and 0.87 for 41°C vs. 35°C, 41°C vs. sham and 35°C vs. sham).

### Low internal ethanol (∼7 mM) does not affect olfaction or locomotion

**Figure 3:**
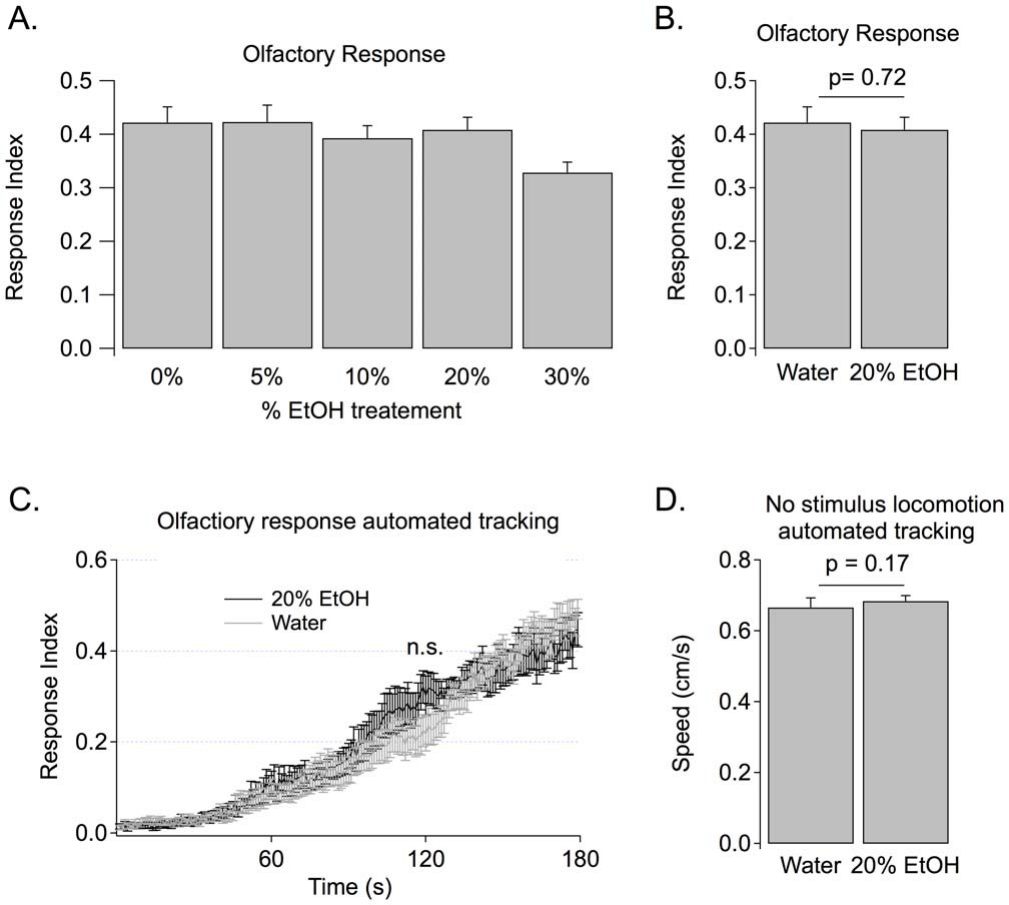
Olfactory response and locomotion are unaffected by ethanol. (**A–C**) Response indices are shown for larvae when placed in the middle of an agar dish with ethyl acetate on one side and liquid paraffin on the other. The number of larvae in each odor zone was counted after 3 minutes. **A**. The Olfactory response shows a mild reduction with 30% ethanol treatment but not with 20% ethanol treatment. **B**. Larvae had been previously submerged for 20 minutes in either pure water or 20% EtOH. No significant difference was seen. **C**. Automated tracking. Left: Response index over a three-minute period when larvae are being tracked. Larvae had been previously submerged for 20 minutes in either pure water or 20% EtOH. **D**. Average speed in the absence of a stimulus is shown for larvae during the three minute tracking period. 20% EtOH did not cause a significant reduction in either locomotion speed or olfaction.

Ethanol might cause reduced learning because it specifically disrupts learning itself or because it alters sensory perception of either the conditioned stimulus (CS) or of the unconditioned stimulus (US). Given that we used an odor as the CS in our learning assay, we tested larvae for their olfactory response to 10^−4^ ethyl acetate (EA) following 20-minute treatments of 0% (water-only), 10%, 20%, or 30% ethanol. The response index to the odorant was determined by manually counting the number of larvae in proximity of odorant, defined as the odor zone (see methods) and the rest of Petri dish at the end of 3 minutes. Only the 30% ethanol-treated group showed a reduction in the olfactory response index, (Figure 3A, 30% vs. water, 5%, 10%, and 20% ethanol had p = 0.013, 0.022, 0.052, and 0.018 respectively; n = 16, 9, 9, 22 and 13 for water, 5%, 10%, 20% and 30% ethanol respectively), although, statistical significance was lost when a Bonferroni correction was applied. However, because we were interested in studying the consequences of an ethanol dose that perturbs higher-order functions without disturbing motor and sensory functions, we chose the 20-minute treatment with 20% ethanol in all further experiments. The 20% ethanol-treated larvae and the water-only group responded equally to 10^−4^ EA (Figure 3B, n = 22, p = 0.72).

Manual end point response measurements can hide differences in the rate of entry to the odor zone that are likely to be more sensitive measures of subtle differences in olfactory responses. Thus we used automated tracking to look at rate of entry of control and ethanol-treated larvae [46]. Using the tracks generated by the software, we were able to analyze the larvae's response indices (similar to manual counting) at any given time. Figure 3C shows the odor response curve of ethanol-treated and water-treated larvae over a 3-minute period. The two groups of larvae showed statistically indistinguishable response indices throughout the test (n = 8, p>0.05 for all individual frames). Using automated tracking, we also quantified speed of larval movement in the absence of odor to see if there are any gross defects in larval locomotion due to ethanol exposure. No significant difference in speed was observed due to ethanol exposure (Figure 3D n = 7, p = 0.17).

The similarity in response indices and speed indicated that animals retain the ability to respond to a conditioning experience even when exposed to ethanol. Therefore, it is reasonable to assume that any defects in learning reflect the impact of low-level ethanol on higher order information processing.

### The effect of ethanol on learning is dependent on the intensity of the US

**Figure 4:**
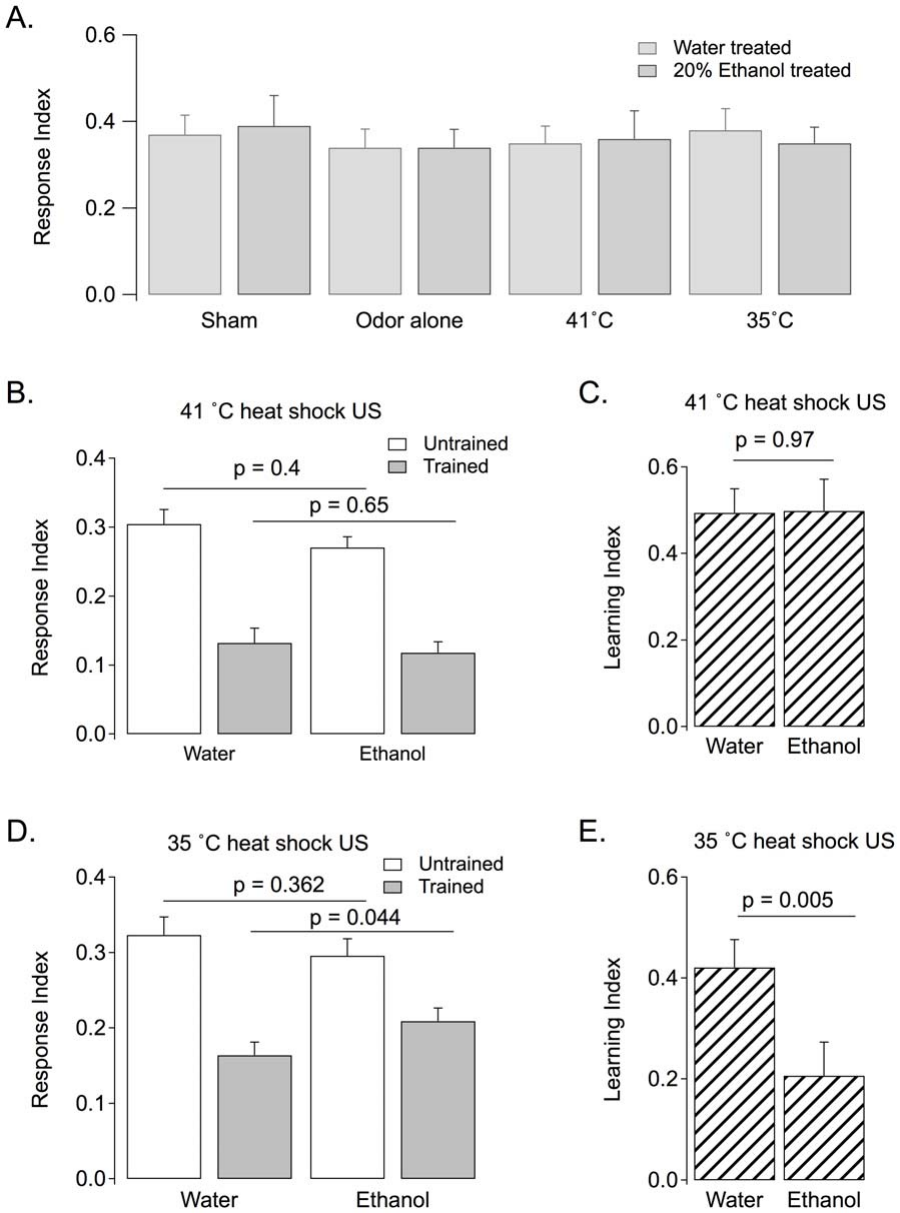
Ethanol treatment affects olfactory learning when the heat shock unconditioned stimulus is below the temperature optima. **A**. Either heat alone or odor alone presentations resulted in the same response index (RI = #Larvae in odor zone/#Larvae total) as sham-treated larvae (p>0.05 for any comparison). **B**. Response indices for untrained (control) and trained larvae are shown for animals that either received water or 20% EtOH. All larvae were trained to associate the odor with a 41°C heat-shock. The response indices were similar for water-treated and ethanol-treated groups when comparisons were made for similar conditions such as the untrained group or the trained group. **C**. Learning indices (LI = (RI_control_−RI_conditioned_)/RI_control_) calculated from the data in Panel B. **D**. Response indices for untrained (control) and trained larvae are shown for animals that either received water or 20% EtOH. All larvae were trained to associate the odor with a 35°C heat-shock. The conditioned response indices are significantly different in the ethanol treated groups (n = 32; p = 0.044). **E**. Learning indices calculated from the data in Panel D. Ethanol induced a significant reduction in learning.

To explore the effect of ethanol on learning, we paired an odor with a heat shock and compared olfactory responses of this trained group with an untrained group. The learning index was calculated as the fractional decrease in olfactory response from the control group following conditioning [(Response Index_(control)_−Response Index_(conditioned)_)/Response Index_(control)_]. We confirmed that, regardless of the ethanol or water treatment, larvae exposed to the CS or the US alone had the same olfactory responses as sham-conditioned animals (Figure 4A). Because of this, we used one control group for the remainder of the study, the heat only stimulus (US), in addition to the trained group that received the simultaneous odor-heat (CS-US) pairing. The ethanol treatment did not alter the odor response of the untrained larvae (immediately after ethanol exposure; Figure 3 A to C) when the internal ethanol concentration was ∼7 mM nor when the internal ethanol concentration was ∼2.5 mM (at the time of the end of the training session; Figure 4A).

The optimal punishment temperature for this learning paradigm is 41°C [32]. Here we treated larvae with 20% ethanol and then trained the larvae in our heat shock paradigm by pairing 10^−4^ EA with a 41°C heat shock. After 3 training trials, we found that larvae treated with ethanol learned similarly to larvae that received a water-only treatment (n = 17, p = 0.97). For both treatment groups, the trained larvae responded to the odor significantly less than the control larvae, indicating that learning has occurred. Trained and control response indices were similar for the water- and ethanol-treated larvae (n = 17, p = 0.40 for control and n = 17, p = 0.65 for trained response indices; Figure 4B) and learning indices for the two groups were nearly identical (n = 17, p = 0.97; Figure 4C).

These results suggest that an internal ethanol concentration of ∼7 mM has no effect on larval learning. However, other learning and memory studies have shown that the effects of ethanol on learning become apparent when suboptimal conditioning parameters are used [48], [49]. To test the hypothesis that different learning conditions will reveal effects of ethanol on learning, we tested ethanol-treated larvae in the same paradigm, using a lower heat shock temperature. We found that ethanol-treated larvae had a significantly lower learning index than water treated larvae when trained with a 35°C heat shock (Figure 4D and 4E). Figure 4D shows the trained and control response indices for the ethanol and water treated groups. While the control response indices of the ethanol- and water-treated groups were similarly high (n = 32, p = 0.362) the trained response index of the ethanol group was higher than that of the water group (n = 32, p = 0.044). This resulted in the ethanol-treated larvae having a significantly lower learning index than water-treated larvae (Figure 4E; n = 32, p = 0.005).

### Decreased learning is not caused by anesthetic properties of ethanol

One possible explanation for a decrease in learning at 35°C is that ethanol anesthetizes the larvae to reduce the aversive properties of the heat pulse. This would make the heat shock a less effective unconditioned stimulus. We therefore compared the aversion of larvae to heat before and after ethanol exposure in a manner adapted from Rosenzweig *et al.* [47].

**Figure 5:**
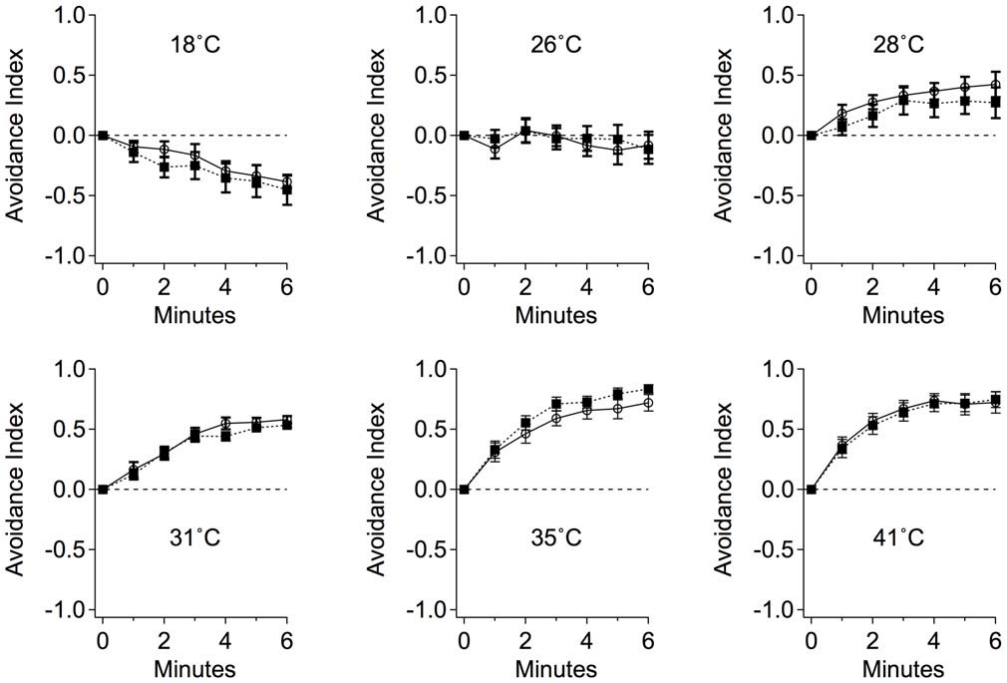
The learning deficit is not caused by ethanol anesthesia. The ethanol treatment did not reduce the sensitivity of larvae to the heat treatment (US). Larvae were placed onto an agar dish in which half of the dish is 24°C and the other half is held at a different temperature. An avoidance index was calculated based on how many larvae avoided the artificially heated or cooled half of the plate. Shown are plots indicating the avoidance index at every minute for the total duration of heat-avoidance assay. The dotted lines with filled squares are ethanol treated group and solid lines with empty circles are the water treated group. The ethanol and water treated groups are not different for any temperature tested (p>0.05 for all points).

We compared the capacity of untreated and ethanol-treated larvae to sense a wide range of temperatures (18°C to 41°C). One half of a Petri dish was cooled or heated and larvae were placed in the center of the dish. Larvae quickly sense the temperature gradient and move towards the side that is closer to their preferred temperature. We calculated the fraction of larvae on the room temperature side of the plate during a six-minute assay. With heat blocks set to produce agar temperatures of 41°C, 35°C, 31°C, 28°C, 26°C or using a cooled block to produce 18°C agar, we observed that the untreated and ethanol treated larvae partitioned similarly between the temperature extremes. The capacity to sense and avoid temperature extremes was not affected by the ethanol treatment (Figure 5, n = 20, p>0.05).

### The effects of ethanol exposure are transient

**Figure 6:**
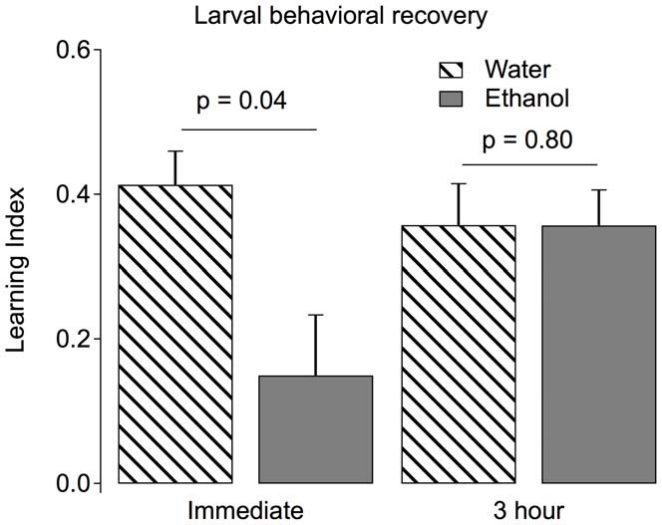
Effects of ethanol are temporary. Behavioral recovery of learning ability after ethanol exposure was tested by dividing both the ethanol-exposed and water-exposed groups into two subgroups, one conditioned immediately after treatment and one conditioned three hours later. No significant difference was observed in the conditioning scores of alcohol and water treated groups at the end of three hours in contrast to the immediately-conditioned group (p = 0.8, n = 5).

We wanted to know if the acute effects of ethanol were permanent or transient. To test this, we conditioned larvae three hours after ethanol exposure. We found that the learning indices of larvae measured three hours following a 20 minute 20% ethanol exposure were statistically indistinguishable from larvae tested three hours following a water exposure (Figure 6). The complete reversibility of the learning deficit indicates that this ethanol treatment does not compromise learning because it induces permanent damage.

## Discussion

In this paper, we show that ethanol negatively affects heat shock induced olfactory associative learning in Drosophila larvae. The deficit in learning is not caused by a deficit in locomotion or olfaction because the drug does not affect odor response indices or speed. Neither is the learning deficit a product of the anesthetic effect of ethanol, since this ethanol treatment did not reduce the response of the larvae in the heat avoidance assay. Thus, the deficit in learning must reflect a subtle perturbation of the learning process by a low dose of ethanol.

This is one of the first Drosophila behavioral assays to capture an effect of low doses of ethanol on learning and memory. Low doses of ethanol, which are commonly thought to be harmless, frequently cause occupation-based injuries or deaths [8], [9]. For the study of the effects of low doses of ethanol, fruit fly larvae are advantageous because they sport all of the genetic tools of Drosophila and have a simple nervous system (∼2000 functional neurons as opposed to ∼100,000 in the adult fly) [50] that lends itself to genetic dissection. Finally, subtle changes in behavior can be quantified because the behavior of large populations of larvae can be quantified using simple and inexpensive methods.

An ethanol-dependent learning deficit is observed when the heat-shock reinforcing temperature is below the optimal temperature for learning. We find a deficit in learning when larvae are heat-shocked at 35°C, but not at 41°C. We suspect that the effects of ethanol are not strong enough to suppress associative conditioning to a strong 41°C heat shock, but can influence a less salient 35°C heat shock. We believe that the use of a sub-optimal US shifts the assay to a region of the stimulus-dependent learning curve that is better suited to reveal the subtle effects of ethanol [32]. In rats, low-level ethanol has been shown to reduce the capacity for attention [23]. In larvae, a diminished capacity for attention could reduce learning by further lowering the effectiveness of the suboptimal US. A lack of visible reduction in learning at 41°C could also be the result of overtraining caused by an ethanol-induced increase in the valence of the punishment at this temperature.

We quantified the internal amount of ethanol in this study using gas chromatography. It is interesting to note that when placed in 20% (3.425 M) ethanol for 20 minutes, the concentration of ethanol within the larvae rises to only ∼7 mM. In humans 7 mM ethanol, which corresponds to a blood alcohol concentration (BAC) of ∼0.03, is considered to be a rather low dose of ethanol. This is a level at which it is currently legal to drive throughout the United States. In larvae, the slow metabolism of ethanol cannot account for the differential between external and internal ethanol concentration (Figure 2D) indicating that the absorption of ethanol by larvae is somehow severely restricted. This is unsurprising, as the natural habitat of larvae includes fermenting fruits that can contain over 7% ethanol [44]. If there were no system to control ethanol absorption then the ethanol that larvae encounter in their natural life cycle would likely be fatal. This capacity to limit ethanol absorption is shared by another invertebrate inhabitant of fermenting fruit [51], [52]. *C. elegans* also show a remarkable ability to limit ethanol absorption when placed in a high ethanol environment. Each model organism provides a unique perspective on a biological process. This paper establishes the use of the genetically malleable fruit fly as a model to study ethanol-induced effects on higher order behaviors like learning.
